# Mr. Peck on Quackery

**Published:** 1801-08

**Authors:** Thomas Peck

**Affiliations:** Higham-Ferrers


### *To the Editors of the Medical and Phyfical Journal.*

GENTLEMEN,

ALTHOUGH many pertinent remarks have been offered to the medical world (through the medium of your valuable Mifcellany) on the fubject of Empiricifm, I almoft fear it is lefs confidered than it ought to be. *Some* plan for the fuppreffion of thefe infidious pretenders is rigoroufly demanded, in *juftice to the community*, as well as the *intereft* of *regular* practitioners.

That the common practice of vending quack medicines has proved highly deleterious to fociety, impaired habits and exhaufted perfons too decidedly indicate; and fo long as thofe fraudulent impoftors are permitted, *unmolefted*, to hurl their rhodomontade among the credulous, fo long will the delufion continue. The advertifing part of thofe infallible trickfters are daily increafing their depredations, are hourly impofing on the community noftrums of nullity or nonfenfe, and with dauntlefs effrontery are procuring patents for what is in *common* ufe in every apothecary's fhop.*

It is true that the *revenue* may in fome meafure be benefited, in confequence of the numerous quantity of ftamps iffued by the venders of medicine; but, that mankind in general fhould continue to be plundered of their property and health, in order to enhance the yearly profits of government, is furely not confiftent with reafon, or compatible with found policy.

Independent, however, of *this* order of quacks, there are others, who, perhaps, are equally (if not more) obnoxious to fociety; profeffing themfelves infallibly capable of diftinguifhing difeafes by a flight infpection of their patient's urine. This clafs (not unaptly denominated Urinarii) is not very numerous; and it would be fortunate for mankind did their impoftures only average their number. They feem to have leaft honor in their own country, though, even at home, there are many whofe credulity expofes them to *empirical ravage*.

Strange as it may appear, it is incontrovertible, that inftances are not wanting, where emiffaries are regularly fent abroad,

---

* Witnefs Mr. Ching's celebrated worm medicine.

with urinals, to collect the *significant* ftreams of the undifcerning afflicted, which meffengers as conftantly forward the *mafs* or *ingredients* to the poor deluded fufferers! Thus, gentlemen, is a liberal profeffion fhamefully undervalued, while fociety at large is notorioufly impofed upon.*

To point out a malady without promifing a remedy, will avail little to the fufferer; I therefore give it as my decided opinion, that nothing fhort of an interference of the legiflative power is equal to the intention of reftraining the evil, is alone capable of counteracting its mifchievous effects, and thereby of promoting the general good of fociety. I further add, that, (as the fubject is of confiderable moment to the regular practitioner) in order to accomplifh an object fo defirable, the neceffary fteps towards fuch an end fhould be purfued immediately.

That a practice fo ignoble fhould continue to be fupported by a country, whofe boaft is to live in an enlightened age, and which abounds with medical talents who do no fmall credit to the day in which they practice, is, to the confiderate mind, a matter of furprife; that it is becoming more and more extenfive, and *ferioufly* prejudicial to the interefts of the faculty (as well as increafingly detrimental to the good of our fellow-creatures) is very evident; and, that it deferves their moft fober regard and determined oppofition, muft ftrike all who have the fmalleft refpect for their own profperity, or the reputation of a liberal profeffion. Should the end not be anfwered by any unforefeen caufes prefenting themfelves, I am perfuaded a ferious reflection on the dignity of the medical character, and a confequent endeavour to thwart every means tending to degrade it, will not be undeferving of the notice of my brethren. *Animam liberavi.*

*Higham-Ferrers,* I am, &c.

*July* 8, 1801. THOMAS PECK.

---

* Many cafes might be adduced, where patients (although convalefcent) have collected fums of money of their neighbours in order to employ an empiric, while their apothecary's ledger teems with unfettled accounts.

Case

